# Lateral Resistance of Modular CFS Shear Wall Connected with Rectangular Steel Tubes and Its Contribution to Frame Structures

**DOI:** 10.3390/ma18235257

**Published:** 2025-11-21

**Authors:** Yanbo Kang, Jiyuan Mei, Xinyu Wu, Liping Wang

**Affiliations:** 1 State Key Laboratory of Building Safety and Built Environment, Beijing 100013, China; kybcabr@126.com; 2 School of Civil Engineering, Central South University, Changsha 410075, China; hfutmjy@163.com (J.M.); wuxinyu0423@163.com (X.W.); 3 CABR Technology Co., Ltd., Beijing 100013, China

**Keywords:** modular cold-formed steel shear wall, lateral resistance, simplified model, seismic response, multi-storey frame

## Abstract

Modular lightweight shear walls can not only facilitate easy installation, thereby improving construction efficiency, but also demonstrate potential to enhance the lateral stiffness when applied in frame structures. The aim of this paper is to investigate the effectiveness of a novel modular cold-formed steel (CFS) shear wall connected with rectangular steel tubes on improving the lateral performance of existing frame structures. Based on the test results of the lateral resistance of four full-scale specimens of modular CFS shear walls connected with rectangular steel tubes, the fine model and simplified model of test specimens were respectively established by the SAP2000v26.0.0 software. The performance indices of the yield load, yield displacement, peak load, peak displacement, and ductility factor were compared, and the maximum error of performance indices was satisfactory. The numerical results show that both the fine and simplified models can well simulate the deformation of walls under lateral cyclic loading, while the simplified models substantially simplify the calculation, which is more adaptable to the subsequent analysis of the multi-story building structure. Then, seismic response analyses of a frame with infilled modular walls and another frame without infilled modular walls were performed. The results indicate that, under the same seismic condition, the lateral displacements of the top floor of the six-story frame with infilled modular walls were reduced by 11–71%, and the maximum inter-story displacement angles were reduced by 15–67% compared to the frame without infilled walls. Therefore, it is demonstrated that the infilled modular CFS shear walls can significantly improve the lateral stiffness and the seismic performance of the steel frame structures.

## 1. Introduction

CFS structures have the advantages of light weight, environmental friendliness, and adaptability to industrialized construction. These merits have contributed to their rapid development in building markets [1]. With the introduction of national policies to vigorously develop the steel structure industry and the gradual implementation of the rural revitalization strategy, it is of great significance to actively promote the wide application of CFS structural systems in low-rise residential buildings and multi-story construction.

As an important lateral force-resisting component of structural systems, CFS walls have been extensively studied by scholars both domestically and internationally. Pan [2] and Bara [3] conducted experimental research on CFS walls. The results showed that the failure modes of CFS walls are mostly the buckling of the side columns, which was found to constrain the shear resistance of the walls, and the section size and strength of side columns play an important role in the shear capacity of the walls. Yu et al. [4,5] conducted a series of lateral loading tests on CFS walls with steel sheathing. The experimental results demonstrated that increasing the thickness of steel sheathing does not improve the shear resistance, whereas taking measures to prevent the premature buckling failure of steel studs can effectively enhance both the shear resistance and ductility of such walls. Jiang et al. [6] performed cyclic tests on CFS composite shear walls with the screw connections reinforced using carbon fiber-reinforced polymer. This novel reinforcement method can not only reduce the cracking damage in screw connections, but also significantly enhance the seismic performance. Yu et al. [7] developed a special energy dissipation X-bracing for CFS shear walls. The experimental results showed that the corrugated steel sheet–sheathed shear walls improved the ductility, and the initial stiffness and shear strength were not significantly reduced. Yao [8] investigated the shear performance of CFS clamped thin steel plate shear walls under monotonic and hysteresis loading. The results revealed that the clamped steel plate can improve the integrity of the shear wall and form a tensile strap, thus significantly improving the lateral stiffness and shear capacity. Ye et al. [9] conducted a series of structural vulnerability analyses on a reinforced cold-formed steel (RCFS) shear wall system and a traditional cold-formed steel shear wall system subjected to earthquake hazard in order to investigate their failure mechanisms. The results showed that the collapse resistance of the RCFS shear wall system is effectively enhanced by the second defense, which is provided by a framework integrated by rigid beam–column joints and fixed column bases.

Due to the limitations of test conditions, an increasing number of researchers have adopted the numerical simulation method to analyze the performance of CFS structure systems. However, the difficulty lies in the fact that, when the CFS walls are subjected to cyclic loading, the resulting hysteresis loop exhibits strength and stiffness degradation as well as the pinch effect phenomenon. Usefi et al. [10] reviewed the numerical models for analyzing CFS walls and divided the numerical simulation methods into two groups: (1) the fine micro method, where all possible failure mechanisms can be captured; (2) the macro method, which includes the equivalent brace method, equivalent spring method, fastener based method, and effective strip method. The fine micro models incorporate detailed geometrical configurations, material properties, mesh discretization, contact interactions, and boundary conditions without excessive simplifications. In the study of the equivalent brace method [11,12,13], the sheathing, braces, and screws were replaced by a single equivalent diagonal brace of equal stiffness. The primary advantage of this method lies in its significant reduction in modeling effort and computation time. However, this approach fails to simulate the distributed forces exerted by the sheathing on the boundary studs. Similar to the equivalent brace method, in the equivalent spring method, the lateral resistance strength and stiffness of walls are directly derived from spring elements. Kechidi et al. [14,15,16] defined two uniaxial materials for the spring element to simulate the wall sheathed with steel and wood boards. However, the continuity of the stud along the height direction is not considered in the model, and when considering the P-delta effect, the influence of the gravity load-resisting system on the lateral stiffness had to be excluded. In the fastener-based method, each fastener is considered as a non-linear, radially-symmetric spring element. Ashok and Wang [17,18] used the fastener-based method in their simulation and compared the results with tests, demonstrating good agreement between the finite element model and tests. Xie and Wu [19,20] used the effective strip method, which is generally employed for CFS shear walls with steel sheathing. However, this method can only determine the ultimate load capacity and fails to capture the hysteretic behavior of the model, so its application is limited.

With increasing building height, the demand for enhanced lateral resistance performance in multi-story structures grows significantly, leading to the widespread adoption of steel frame–shear wall structural systems. The research in [21,22,23,24,25,26,27] demonstrates that the incorporation of CFS walls can improve the lateral performance of structural systems. Under earthquake action, the cooperative deformation of the frame and the wall significantly improves the bearing capacity and lateral stiffness of the whole structure.

Hu and Wang [28,29] proposed and investigated a novel fully prefabricated CFS wall connected with rectangular steel tubes. This type of modular lightweight wall can significantly facilitate fast construction and the retrofitting process. The rectangular connecting steel tube serves a dual purpose: it not only acts as a connector between adjacent wall unit modules but also functions as an edge column at regular intervals within the wall, thereby significantly enhancing the stiffness compared to traditional shear walls with only CFS studs. In order to investigate the effectiveness of the novel modular CFS shear wall connected with rectangular steel tubes on improving the lateral performance of existing frame structures, this paper presents a further extension based on the test results of the lateral resistance of four full-scale CFS wall specimens. Firstly, a fine model that can accurately reflect the mechanical performance of the wall was established based on the SAP2000 software. Then, a simplified model that can simulate the stressed skin effect of the CFS wall was also established based on the equivalent spring method. Both the analysis results from the simplified model and the fine model were compared with the test results to verify the effectiveness and rationality of the simplified model. Subsequently, according to the simplified method, a six-story frame model with the infilled modular CFS shear wall was established. The non-linear time history analysis was carried out to obtain the seismic response of the frame with infilled wall. It was further compared with that of the frame without infilled wall to identify the effect of a modular CFS shear wall with rectangular steel tubes on the seismic performance of multi-story frame structures.

## 2. Fine Model of the Modular CFS Shear Wall with Self-Tapping Screw Connection Details

### 2.1. Test Introduction

**Figure 1:**
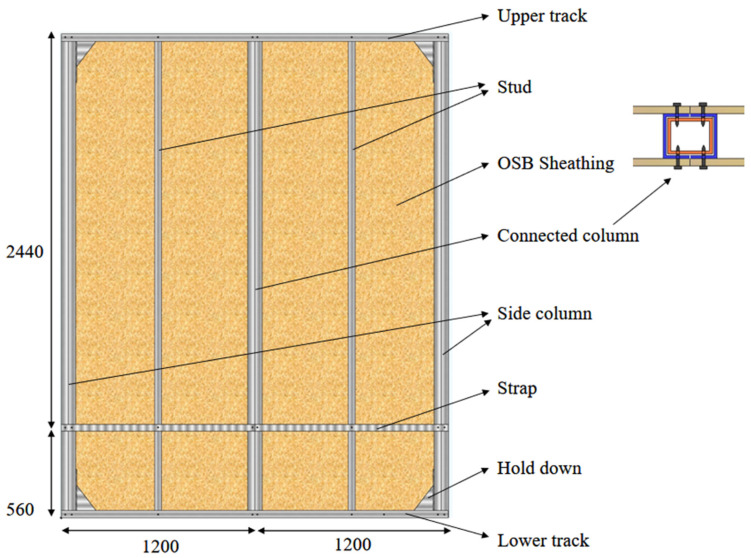
Wall structure.

Four full-scale modular CFS shear walls connected with rectangular steel tube specimens were subjected to lateral low cyclic loading tests, which were previously completed by the authors [28,29]. As shown in Figure 1, the test specimen has a height of 3000 mm and a width of 2400 mm. It consists of CFS skeleton, sheathing, and ST4.2 × 32 self-tapping screws to connect the two.

**Figure 2:**
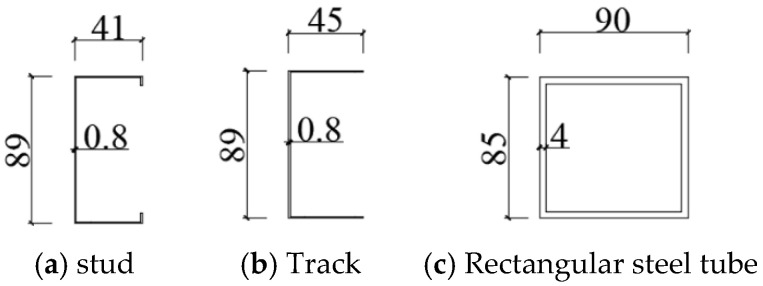
Dimensions of components (unit: mm).

The side column and the connected column of the wall are composed of two U-shaped CFS face-to-face coated rectangular steel tubes. The stud is a single C-shaped CFS, and the upper track and lower track are U-shaped CFSs. The dimensions of the sheathing used in the wall specimens are 2440 mm × 1220 mm and 560 mm × 1220 mm, and the thickness is 11 mm. The numbers and parameters of the wall specimens are shown in Table 1. The sheathing of the wall is made of oriented strand board (OSB), whose modulus of elasticity is 2540 MPa, and the static bending strength is 17.45 MPa. The CFS studs, as shown in Figure 2a, and CFS tracks, as shown in Figure 2b, used in the test have a strength of 550 MPa, and the rectangular steel tube is made of Q355 steel, as shown in Figure 2c. The distance between the peripheral screw is 100 mm, and the internal distance between the screws is 300 mm.

### 2.2. Test Results

**Figure 3:**
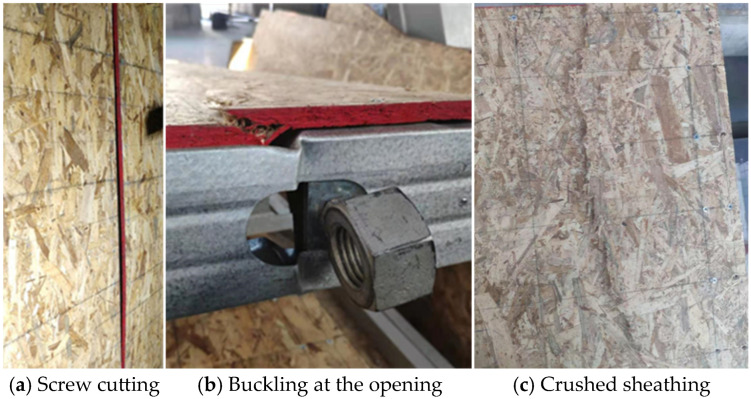
Failure mode.

Specimen damage mainly manifested in two modes. For the single-sided OSB wall specimens, the damage was mainly shear at the vertical screw joints. Eventually, this resulted in the detachment of the sheathing from the steel skeleton, weakening the stressed skin effect, as shown in Figure 3a. There was no damage to the OSB after the test. For the double-sided OSB wall specimens, the damage was mainly caused by the pressure buckling at the opening of the upper track. Finally, the sudden buckling at the opening resulted in the crushing of the sheathing near the upper track, which ultimately led to the damage of the OSB, as shown in Figure 3b,c.

According to the test results in Table 2, the conclusions are as follows: the lateral stiffness of the CFS wall connected with rectangular steel tubes is greatly improved, but the ductility is reduced.

The lateral load capacity of double-sided OSB wall specimens is greatly improved compared with single-sided OSB wall specimens. Reducing the spacing of the steel skeleton from 600 mm to 400 mm can improve the lateral resistance of the wall, but the effect is not significant.

### 2.3. Establishment of Fine Model

#### 2.3.1. Simulation of CFS Studs and Sheathing

In the SAP2000 software, the stud, track, column, and horizontal CFS in the wall are simulated using the 1D frame element. In the test, they are connected by self-tapping screws at the contact part, so it is set to be hinged in the model.

The OSB sheathing is simulated using the shell element. The shell element in SAP2000 is divided into a thin shell element and thick shell element, and the out-of-plane and in-plane stiffnesses are considered. The thin shell element is suitable for the case where the thickness–width ratio of the shell is less than 0.1. However, the effect of the transverse shear deformation on the calculation of deflection is ignored. Since the thickness of OSB in the test is 11 mm, which is much smaller than its width, the thin shell element was chosen.

#### 2.3.2. Simulation of Self-Tapping Screws

From the test phenomena and research, it is generally believed that the lateral resistance of the CFS wall is mainly provided by the stressed skin effect formed by the connection of the steel skeleton, sheathing, and self-tapping screws, where the self-tapping screws are mainly subjected to shear force.

**Figure 4:**
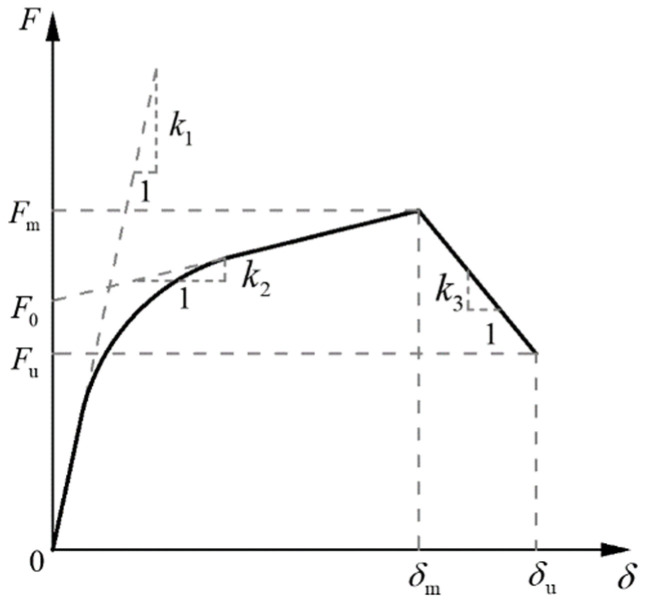
Modified exponential “Foschi” skeleton curve.

The modified exponential “Foschi” equation [30], as shown in Equation (1), is used for modeling the self-tapping screw connections in the wall, and the skeleton curve of the self-tapping screw is shown in Figure 4.

(1) $$F=\left\{\begin{array}{l} \mathrm{sgn}(\delta )\cdot (k_{2}\left|\delta \right|+\left|F_{0}\right|)(1-e^{-k_{1}\left|\delta \right|/F_{0}})\\ \left|\delta \right|\leq \left|\delta_{m}\right|\\ \mathrm{sgn}(\delta )\cdot (\left|F_{m}\right|+k_{3}(\left|\delta \right|-\left|\delta_{m}\right|)\\ \left|\delta_{m}\right|<\left|\delta \right|\leq \left|\delta_{u}\right|\\ 0\;\left|\delta \right|>\left|\delta_{u}\right| \end{array}\right.$$

The six control parameters of the modified exponential “Foschi” skeleton curve are shown in Table 3.

**Figure 5:**
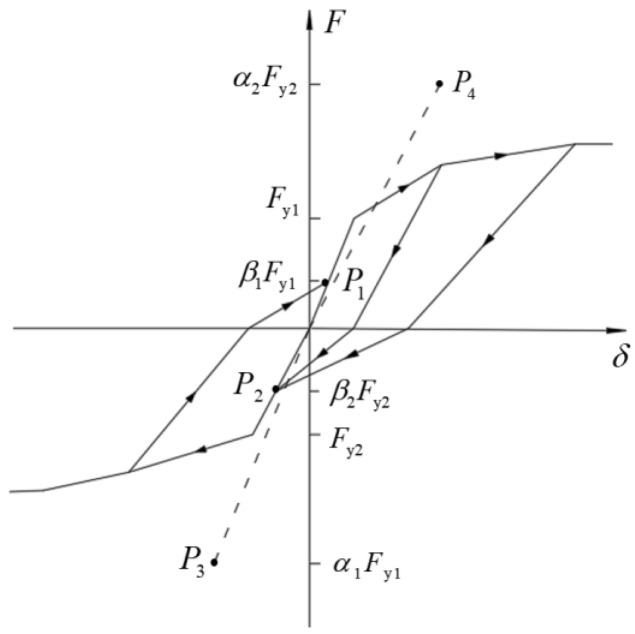
Pivot hysteretic model.

In the SAP2000 software, the multi-linear plastic connection element is used to simulate the self-tapping screw in the wall, and the hysteretic characteristics of the self-tapping screws are based on the pivot hysteretic model, as shown in Figure 5. By controlling the values of α_1_, α_2_, β_1_, and β_2_, the pinching characteristics of the hysteresis curve of the self-tapping screw connection can be simulated. The control parameters of the pivot hysteretic model are determined to be α_1_ = α_2_ = 40, β_1_ = β_2_ = 0.6.

#### 2.3.3. Boundary Conditions and Loading

The setting of the boundary conditions of the wall is based on the actual test device. Fixed constraints are applied to the nodes at the lower track to constrain their degrees of freedom in the U_1_, U_2_, U_3_, R_1_, R_2_, and R_3_ directions. We apply constraints to the nodes at the upper track to constrain their out-of-plane degrees of freedom.

In the test, lateral low cyclic loading was carried out at the top of the wall using two steel plates connected by a long rod as a loading transfer device, so that lateral displacement was applied to the nodes at the top of the wall at both edge regions. The simulation of lateral low cyclic loading of the wall is achieved by setting the time history analysis loading condition. The displacement loading of the model is controlled by the same load steps as the test loading, with a displacement load of 2 mm in cyclic increments per stage.

**Figure 6:**
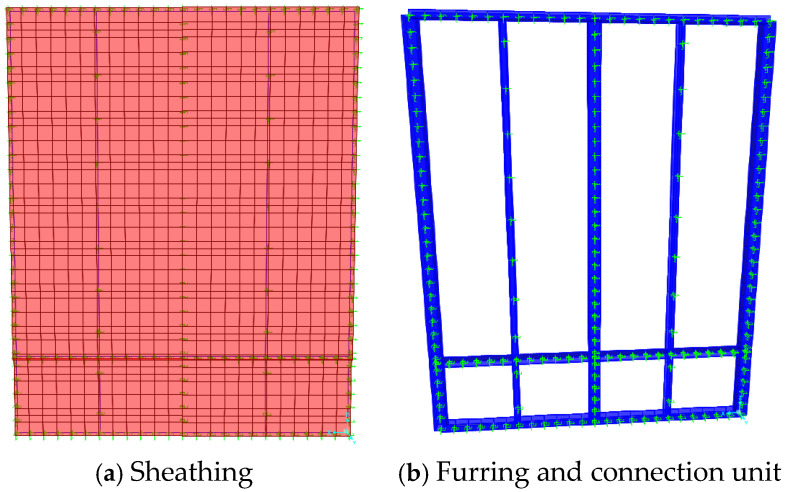
Fine model of specimen W-600-OS.

Figure 6 shows a schematic diagram of the model of W-600-OS after the completion of the building according to the above steps.

### 2.4. Comparison of Results Obtained from the Fine Model and Test

#### 2.4.1. Comparison of Deformation of the Modular Wall

**Figure 7:**
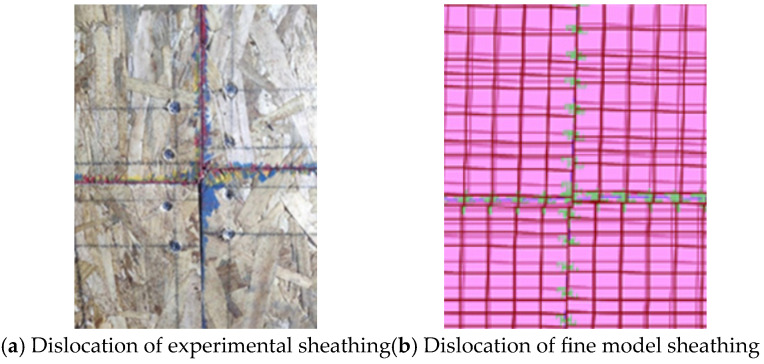
Comparison of dislocation between test and fine model.

**Figure 8:**
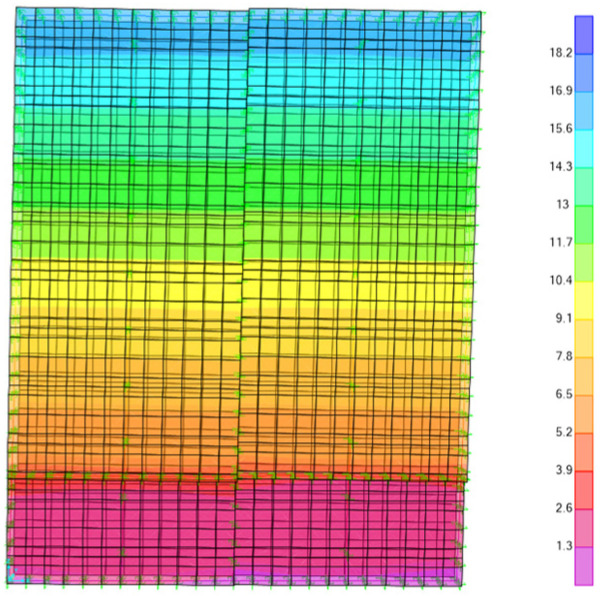
Lateral displacement of fine model.

Comparing Figure 7a,b, it can be seen that the fine model shows the phenomenon of sheathing misaligning and squeezing during the lateral low cyclic loading, which is consistent with the test. The lateral deformation pattern of the fine model is also consistent with the test. As shown in Figure 8, the lateral displacement decreases gradually from the top to the bottom of the wall.

#### 2.4.2. Comparison of Hysteretic Curve and Skeleton Curve from the Fine Model Analysis

**Figure 9:**
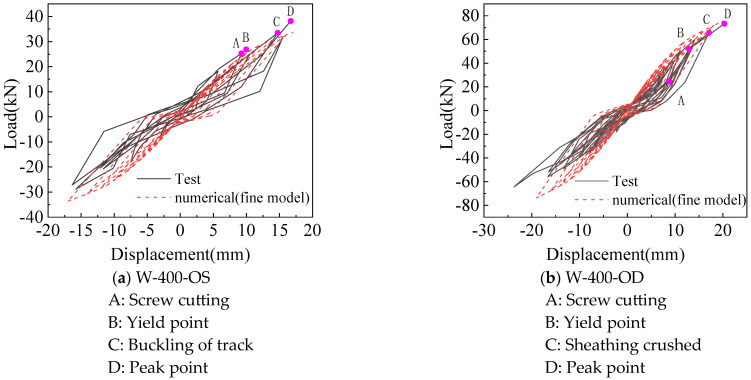

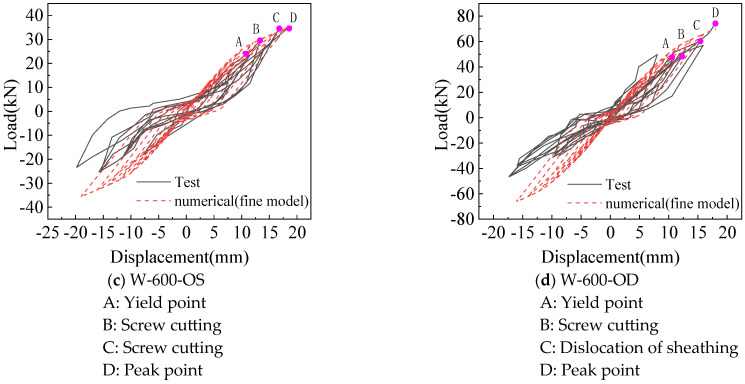
Comparison of hysteretic curves between test and fine model.

Comparison of the hysteretic curve between the fine model and test is shown in Figure 9. The model can better simulate the stress performance of the CFS wall under lateral low cyclic loading. At the early stage of loading, the fine model is in the elastic stage as the test wall, and enters the plastic stage as the load increases. The hysteretic curve takes on a bow shape, reflecting the obvious characteristics of the pinch effect.

**Figure 10:**
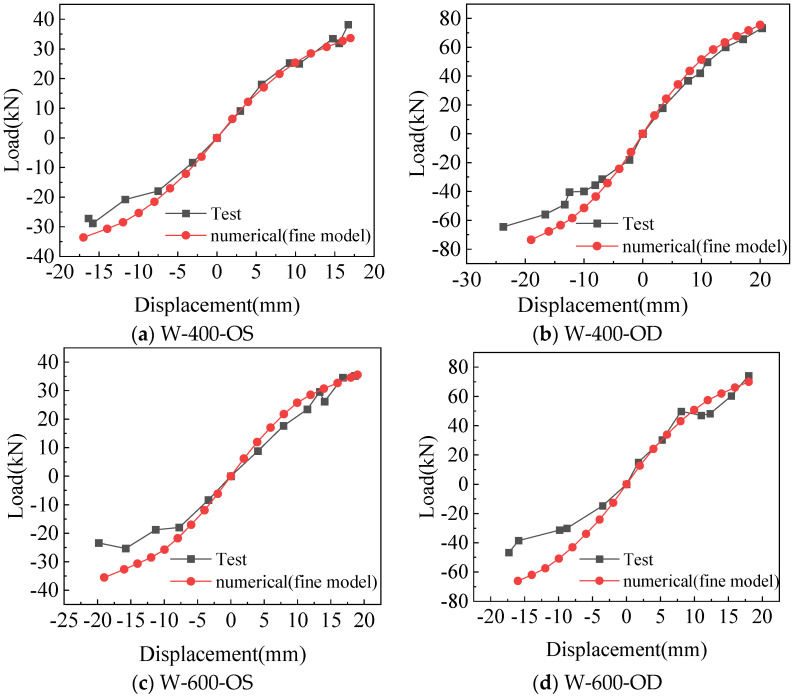
Comparison of skeleton curves between test and fine model.

Comparison of the skeleton curve between the fine model and test is shown in Figure 10. There is no obvious yield point and yield stage in the skeleton curves obtained by simulation and test, and they are discontinued after reaching the peak point. For positive loading, the simulated and tested skeleton curves are in good agreement at the beginning of loading, and the peak loads are also close to each other. For negative loading, the simulated and tested skeleton curves match well at the beginning, and the error increases with the increase in negative loading. Generally, the simulated skeleton curves are in good agreement with the test.

#### 2.4.3. Comparison of the Performance Indices Between Test and Fine Model 

Comparison of the performance indices between the fine model and test are shown in Table 4. The error between the yield loads of the fine model and the test ranged from −9.17% to 1.44%, and the yield displacements of fine model are smaller than the test, with an error of −9.34% to −12.48%. The error between the peak loads of the fine model and the test ranged from 2.91% and −11.86%. The error in peak displacement ranged from 1.38% and 2.91%. Due to the error of the yield displacement obtained by the simulation, the error between the consequent calculated ductility factor obtained by the simulation and the test is 8.33% and 16.22%.

The reason for the difference in yield displacement is that there is a discrepancy between the constitutive model of the self-tapping screw in the simulation and the actual performance of the screw used in the test. Furthermore, in the simulation, the bottom boundary condition of the model is fixed, which is stronger than the actual situation in the experiment. In addition, due to the rectangular steel tubes restraining the swing of the self-tapping screws at the side column and the middle column during the loading process, the lateral stiffness of the wall is improved while reducing the horizontal displacement of the wall. As a result, the damage form of the self-tapping screws at the middle column of the wall was changed to shear failure, which led to the damage of the wall occurring suddenly, resulting in the reduction in the ductility factor of the wall.

## 3. Simplified Model of the Modular CFS Shear Wall Using Equivalent Spring Method

Due to the high connections of self-tapping screws in the CFS wall in this paper, too many non-linear connection elements will lead to large calculations and intricate analyses. In this section, based on the equivalent spring method to simulate the stressed skin effect of the CFS wall, simplified models were established in SAP2000 for the non-linear analysis.

### 3.1. Equivalent Spring Method and Establishment of Simplified Model

In the equivalent spring method, it is generally believed that the lateral resistance of the CFS wall is mainly provided by the stressed skin effect formed by the connection of the steel skeleton, sheathing, and self-tapping screws. In this approach, the steel skeleton, sheathing, as well as the screws are simulated by a single equivalent spring; the lateral resistance of the wall is derived directly from the equivalent spring element.

**Figure 11:**
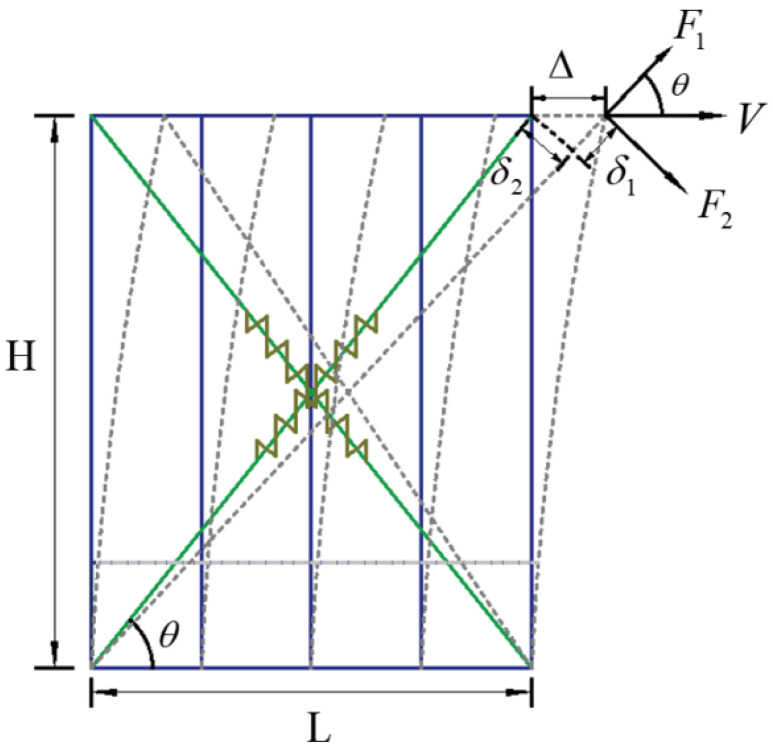
Equivalent spring model of wall.

The equivalent spring model is shown in Figure 11, where the wall skeleton is simulated by a hinged rod element, and the sheathing plates are equivalent to two non-linear spring elements. They follow the pivot hysteretic rule and are simulated by the multi-linear plastic connection elements in SAP2000. The simplified method only considers the horizontal lateral force provided by the stressed skin effect, ignoring the vertical shear force when subjected to vertical load. The control parameters of the equivalent spring element are determined to be *K*_1_ = 4784 N/mm and *K*_2_ = 2109 N/mm, and the resulting lateral force and displacement of the CFS wall are as follows:

*F*_1_ = 1/2 · *V* · cos *θ* (2)

*δ*_1_ = ∆ · cos *θ* (3)

*K*_1_ = *F*_1_*/δ*_1_ = *K* (4)

*F*_2_*=* 1/2 · *V* · sin *θ* (5)

*δ*_2_ = ∆ · sin *θ* (6)

*K*_2_ = *F*_2_*/δ*_2_ = *K* (7)

### 3.2. Comparison of Results Obtained from Simplified Model and Test

The comparison of results in this section is divided into three aspects, including the hysteretic curve, skeleton curve, and performance indices.

#### 3.2.1. Comparison of Hysteretic Curve and Skeleton Curve from the Simplified Model Analysis

**Figure 12:**
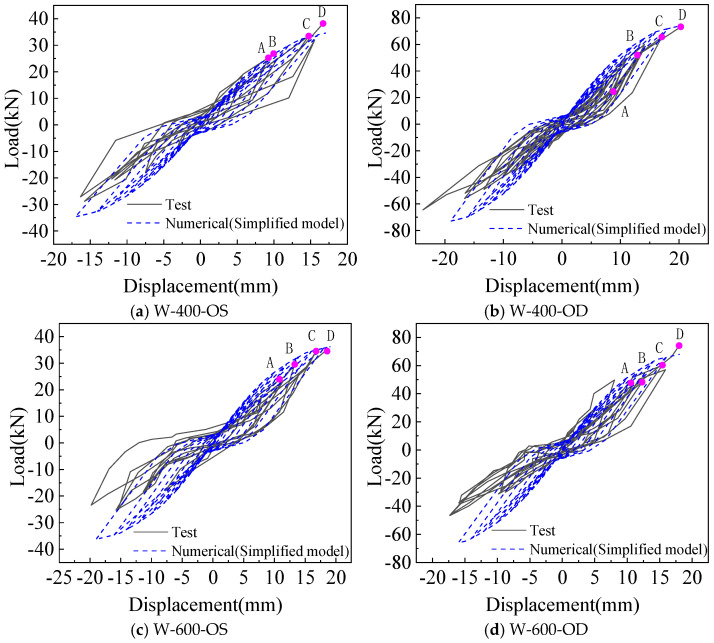
Comparison of hysteretic curves between test and simplified model.

Comparison of the hysteretic curve between the simplified model and the test is shown in Figure 12, where the hysteretic curve calculated by the simplified model can simulate the pinch effect in the test.

**Figure 13:**
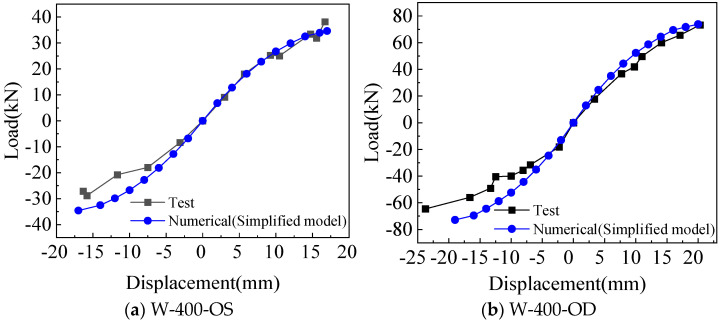

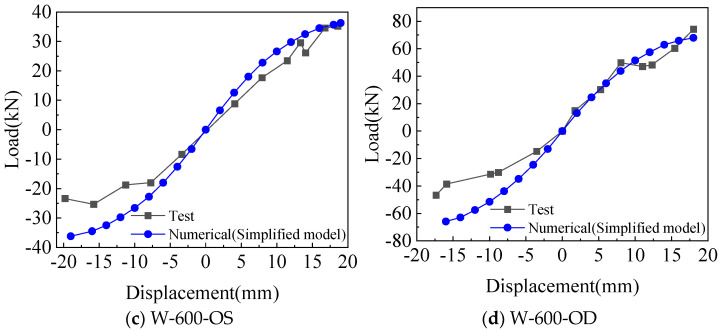
Comparison of skeleton curves between test and simplified model.

Comparison of the skeleton curve between the simplified model and the test is shown in Figure 13. W-400-OS, W-400-OD, and W-600-OD show better matches with the tests in the positive loading, with the peak loads being closer to each other, and the error under negative loading is slightly larger than that under positive loading. The peak load of the skeleton curves obtained from W-600-OS calculations is in good agreement with the test, but the initial stiffness is slightly greater than the test.

#### 3.2.2. Comparison of the Performance Indices Between Test and Simplified Model 

Comparison of the performance indices between the simplified model and the test is shown in Table 5. The yield loads calculated by the simplified model are closer to the test, with a maximum error of 5.87%. The yield displacements of the simplified model are smaller than the test, with an error of −14.24% to −16.22%. The error between the peak loads of the simplified model and the test ranged from −9.32% to 8.38%, whereas the peak loads of W-400-OD are in better agreement with the test, with an error of 0.80%. The error between the ductility factor calculated by the simplified model and the test ranged from 0.67% to 3.5%, which is greater than the test.

### 3.3. Comparison of the Results Between the Fine Model and the Simplified Model Analysis

**Figure 14:**
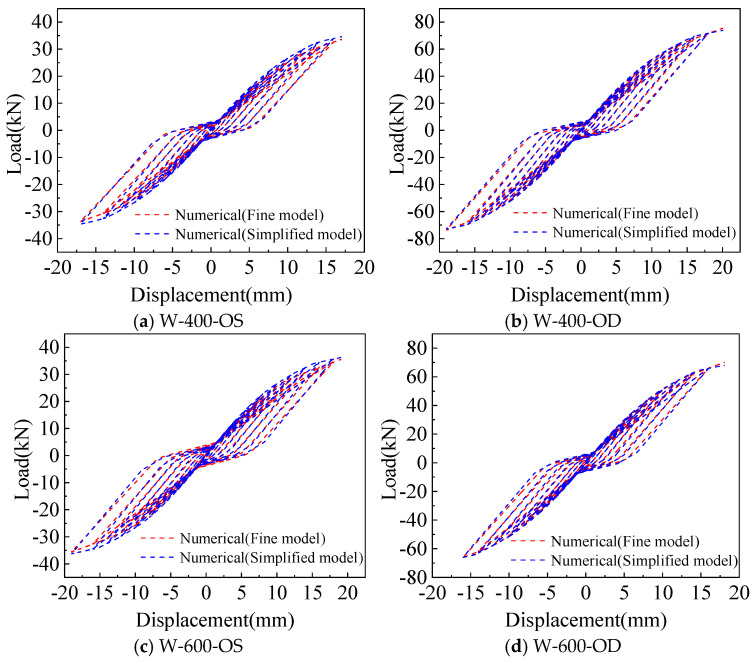
Comparison of hysteretic curves between fine model and simplified model.

**Figure 15:**
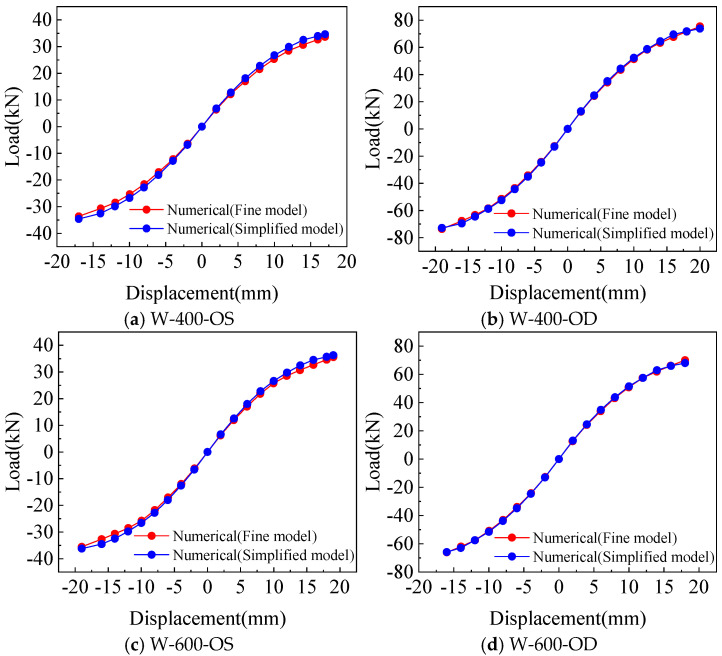
Comparison of skeleton curves between fine model and simplified model.

As shown in Figure 14 and Figure 15, comparing the calculation results of the fine model based on the screw connection and the simplified model using the equivalent spring method, it can be found that the hysteretic curve simulated by the two models can reflect the pinch effect of the wall. The yield and peak load, as well as yield and peak displacement, calculated by the two models are in good agreement with the test. Therefore, both the fine model and the simplified model can accurately simulate the mechanical properties of a CFS wall connected with rectangular steel tubes, but the accuracy of the simplified model in the yield displacement and ductility coefficient of the wall is slightly lower than that of the fine model.

Moreover, the equivalent spring method simplifies the modeling process, and also reduces the difficulty and time of calculation, making it easier to apply to the analysis of multi-story building structures.

## 4. Seismic Response Analysis of Multi-Story Building Structures Based on the Collaborative Work of Modular Walls and Frames

According to the simplified analysis method of the aforementioned wall, this section will explore the difference in the seismic performance of the CFS wall with rectangular steel tubes applied to the overall house frame structure, forming a CFS wall–frame structure system, and study its seismic performance.

### 4.1. Establishment of Multi-Story Building Model

#### 4.1.1. Engineering Background and General Introduction

**Figure 16:**
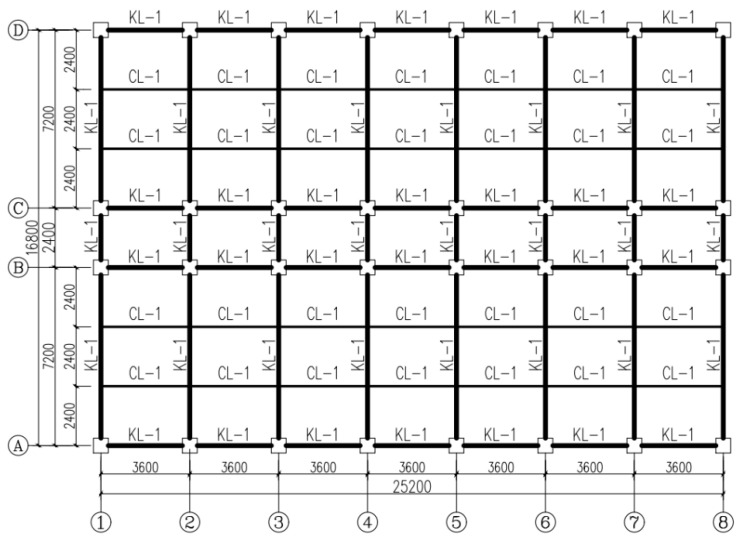
Structural layout plan (unit: mm).

**Figure 17:**
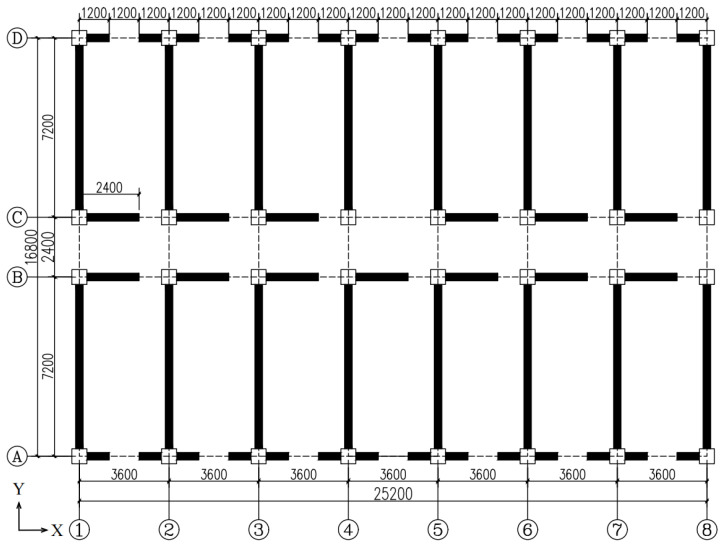
Wall layout plan (unit: mm).

In this study, two engineering cases of a frame without infilled wall and a frame with infilled wall were designed for the simulation. The seismic fortification intensity was eight degrees, and the design value of basic seismic acceleration was 0.20 g. The structural type was a steel frame with a total of six floors, where the height of every floor was 3 m and the total height was 18 m. The floors (roofs) were made of 150 mm thick cast-in-place concrete, and the concrete of the floors (roofs) was C30. The beam and column arrangement is shown in Figure 16. The section of the main beam (KL-1) is HM300 × 200 × 8 × 12 mm, and the section of the secondary beam (CL-1) is HM250 × 175 × 7 × 11 mm. The section of the column is 300 × 300 × 10 × 10 mm. The steel frame columns and beams were all made of Q355. The wall layout is shown in Figure 17. W-600-OD is selected for the infilled CFS wall. Since CFS walls with holes are not studied in this paper, the walls are not arranged where door and window openings are required.

#### 4.1.2. Establishment of Models and Selection of Parameters

The beam and column are simulated by rigid connecting rod elements, and the plastic hinges are arranged in the region of the main beam at a relative position of 0.1 from the ends. The floor is simulated by a shell element based on the assumption of a rigid floor. This leads to the fact that the modal shape of the structure is mainly determined by the stiffness distribution of the frame, Also, this means that the calculation of inter-story displacement mainly depends on the stiffness and stress of the frame column. Mass lumping concentrates the mass of the structure at the height of the floor. This assumption simplifies the dynamic equation and makes the modal analysis easier. The walls were modeled by using the simplified model in Section 2, which are hinged with steel frames.

The dead load of the floor is 2.0 kN/m^2^, the live load is 2.0 kN/m^2^, and the live load of the roof is 2.5 kN/m^2^. The weight of the wall is equivalent to the line load applied to the corresponding beam, which is 2.93 kN/m. The wall at the opening of the door and window is deducted from the corresponding weight of the door and window, which is simplified to line loads of 1.95 kN/m and 2.44 kN/m. The corresponding line load of the parapet is 1.8 kN/m. For the frame without infilled wall, the same linear load is applied at the same position.

**Figure 18:**
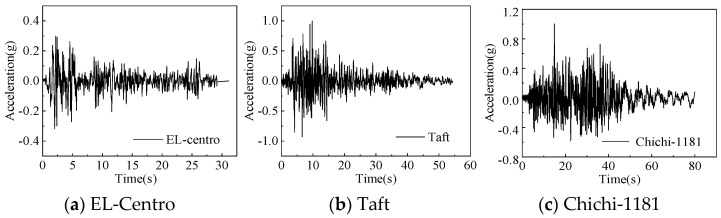
Seismic waves.

The seismic fortification intensity was eight degrees. According to the maximum seismic acceleration for the time history analysis in the code for the seismic design of buildings [31], the peak seismic wave acceleration under frequent earthquake was 0.7 m/s^2^ and the peak seismic wave acceleration under a rare earthquake was 4 m/s^2^. The El-Centro, Taft, and Chichi-1181 waves were input into SAP2000 software, as shown in Figure 18, and seismic acceleration loads were applied to the X- and Y-directions of the two models.

### 4.2. Modal Analysis of the Building Models

**Figure 19:**
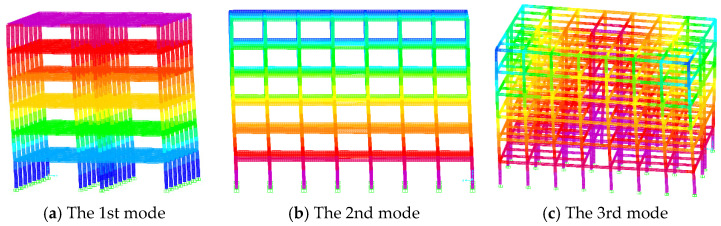
First three vibration modes of frame without infilled wall.

**Figure 20:**
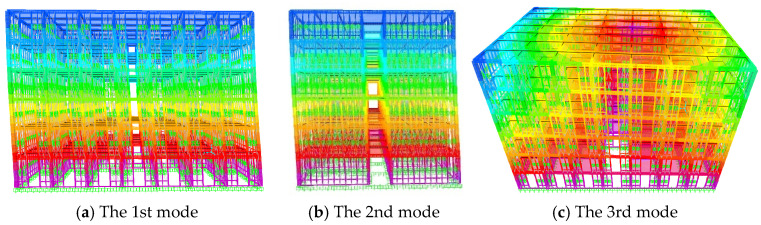
First three vibration modes of frame with infilled wall.

The comparison of the first three natural vibration periods and vibration modes of frame without infilled wall and frame with infilled wall is shown in Table 6. Compared with the frame without infilled wall, the first three natural vibration periods of frame with infilled wall are reduced to a greater extent, which indicates that the stiffness of the wall–steel frame model is greatly improved. Figure 19 shows the first three vibration models of frame without infilled wall. Because of the large spacing of columns in the Y-direction, the stiffness in the Y-direction is low, so that the first vibration model is a translation of the Y-direction. Figure 20 shows the first three vibration models of frame with infilled wall; due to the great number of openings of doors and windows in the X-direction, this results in the lateral stiffness of the X-direction being lower than that of the Y-direction, so that the first vibration model is a translation of the X-direction.

### 4.3. Comparison of Seismic Response of the Frame Buildings with and Without Infilled Wall

The comparison of the main numerical results of the time history analysis, such as structural lateral displacement, inter-story displacement angle, and base shear, are shown as follows.

#### 4.3.1. Lateral Displacement of the Structures

**Figure 21:**
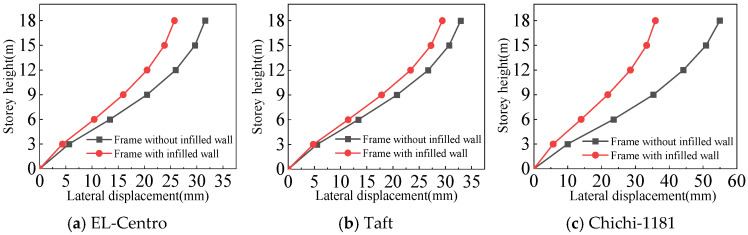
Comparison of lateral displacement under frequent earthquakes in the X-direction.

**Figure 22:**
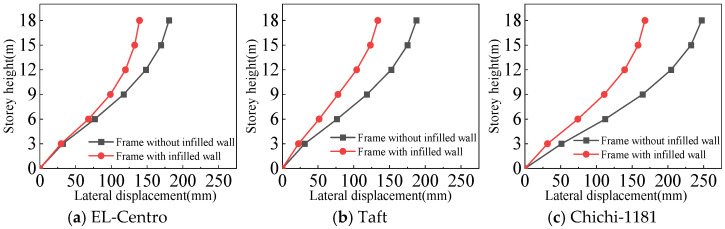
Comparison of lateral displacement under rare earthquakes in the X-direction.

**Figure 23:**
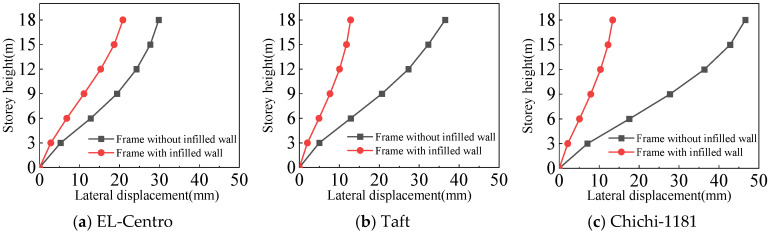
Comparison of lateral displacement under frequent earthquakes in the Y-direction.

**Figure 24:**
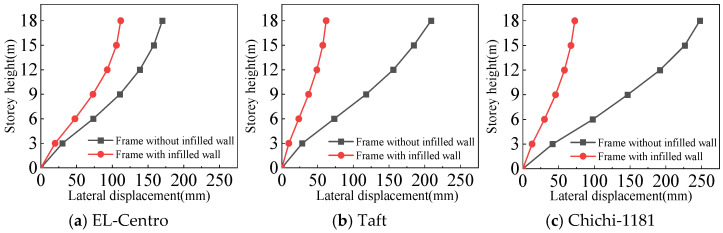
Comparison of lateral displacement under rare earthquakes in the Y-direction.

From Figure 21, Figure 22, Figure 23 and Figure 24 and Table 7, the following can be concluded:

(1) For the frame with infilled wall, because the doors and windows are set in the X-direction, the stiffness of the X-direction of the model is lower. The lateral displacement of the top floor in the X-direction is greater than that in the Y-direction under frequent and rare earthquakes. This corresponds to the fact that the X-direction translational natural vibration period from the structural modal analysis is greater than that in the Y-direction.
(2) Both the frame with infilled wall and frame without infilled wall show shear-type deformation under all three seismic wave time history analyses. Horizontal lateral displacement increases with increasing earthquake intensity for both frames. In the same situation, the lateral displacement of each floor of the frame with infilled wall is always smaller than that of the frame without infilled wall. Comparisons show that the maximum lateral displacements of the top floor of the frame with infilled wall are 11–71% lower than those of the frame without infilled wall, which indicates that the infilled wall increases the horizontal lateral stiffness of the structure.

#### 4.3.2. Inter-Story Displacement Angle

**Figure 25:**
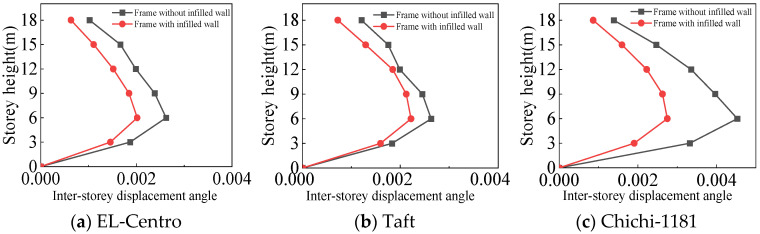
Comparison of inter-story displacement angle under frequent earthquakes in the X-direction.

**Figure 26:**
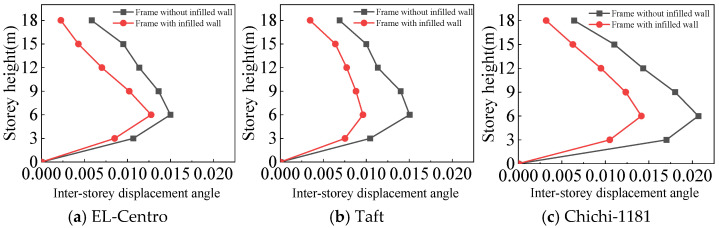
Comparison of inter-story displacement angle under rare earthquakes in the X-direction.

**Figure 27:**
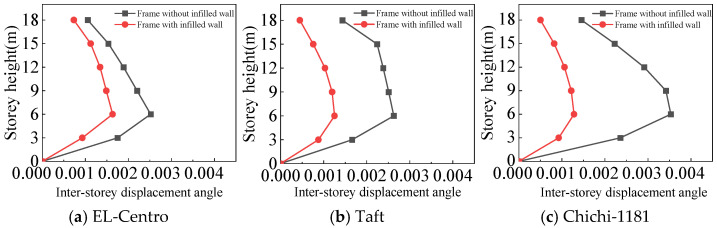
Comparison of inter-story displacement angle under frequent earthquakes in the Y-direction.

**Figure 28:**
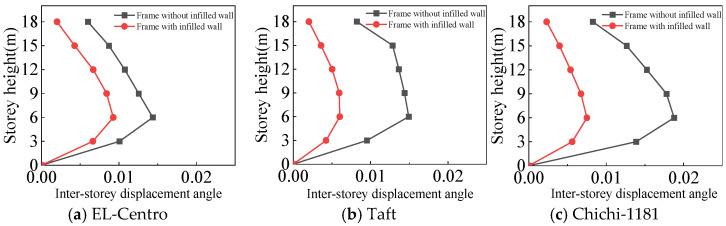
Comparison of inter-story displacement angle under rare earthquakes in the Y-direction.

From Figure 25, Figure 26, Figure 27 and Figure 28 and Table 8, the following can be concluded:

(1) The maximum inter-story displacement angle occurs in the second floor for both frames under frequent and rare earthquakes. This indicates that the second floor is the weak floor for both frames.
(2) The inter-story displacement angle of the frame with infilled wall under the same situation is smaller than that of the frame without infilled wall. The maximum inter-story displacement angle of the frame with infilled wall is 15–67% lower than that of the frame without infilled wall.
(3) The code for the seismic design of buildings [31] stipulates that the elastic inter-story displacement angle limit for multi-story structures under frequent earthquakes is 1/250, and under rare earthquakes it is 1/50. The maximum inter-story displacement angle of the frame with infilled wall is 1/364 under frequent earthquakes, and the maximum inter-story displacement angle of the frame with infilled wall is 1/71 under rare earthquakes, which satisfies the requirements of the code for the seismic design of buildings [31].

#### 4.3.3. Base Shear

From Table 9, it can be seen that the base shear of the frame with infilled wall increases by 35–119% for seismic conditions in the Y-direction compared to the frame without infilled wall, while the base shear in the X-direction shows irregular change under different seismic conditions. This may be due to the fact that the modular shear walls were arranged more continuously along the Y-direction.

#### 4.3.4. Performance Analysis of Wall Components

**Figure 29:**
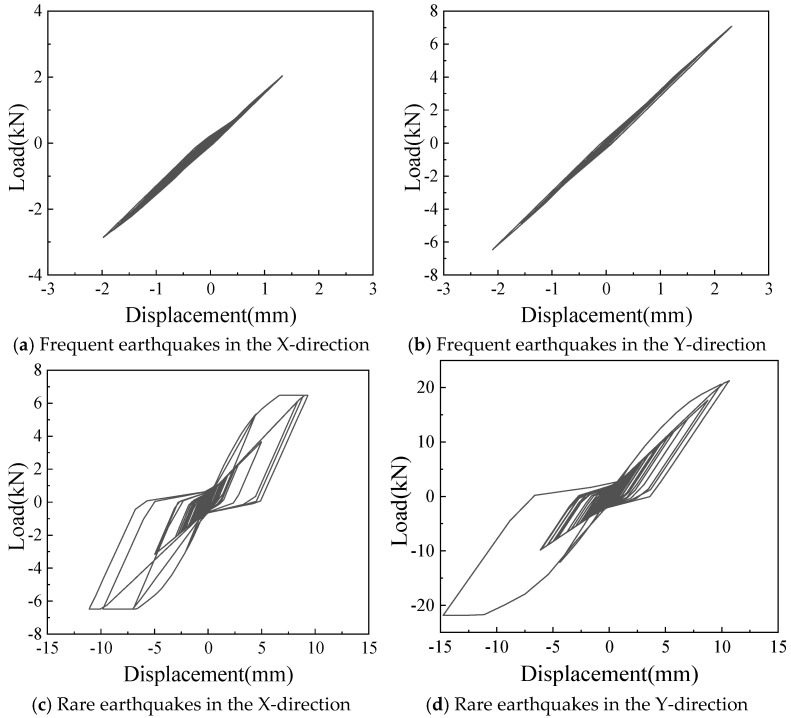
Hysteretic curves of single wall.

As shown in Figure 29, the hysteretic curve of stressed skin effect units in a single wall in the second story of the frame with infilled wall under the seismic condition of the EL-Centro wave is selected. Under frequent earthquake conditions, the stressed skin effect unit is in an elastic state, and under rare earthquake conditions, the stressed skin effect unit is in a plastic state and participates in plastic energy dissipation.

## 5. Conclusions

Based on the cyclic loading tests of four full-scale modular CFS shear walls connected with rectangular steel tubes, fine and simplified numerical models were established and verified, respectively. Two six-story building models were then developed for structural analyses under seismic actions. The main findings of this research can be summarized as follows:

(1) Both the fine model with screw connection details and the simplified model using the equivalent spring method established in this paper can well simulate the deformation of the modular CFS shear wall under lateral low cyclic loading, reflecting the same characteristics of the pinch effect. The yield and peak load, as well as yield and peak displacement, calculated by the two models are in good agreement with the test. Although the calculation accuracy of the simplified model is slightly lower than that of the fine model, it greatly simplifies the calculation and is more suitable for the subsequent analysis of multi-story building structures.
(2) Both the frame with infilled wall and frame without infilled wall show shear-type deformation under the same horizontal seismic force. It is shown that the maximum lateral displacements of the top floor of the six-story frame with infilled wall are 11–71% lower than those of the frame without infilled wall, and the maximum inter-story displacement angles were reduced by 15–67%, which indicates that the infilled wall effectively increases the horizontal lateral stiffness of the structure.
(3) Under frequent earthquake conditions, both of the frames with and without infilled walls are in the elastic state, while under rare earthquake conditions, the modular CFS shear wall is in the plastic state and participates plastic energy dissipation.

## Figures and Tables

**Table 1:** Specimen number and parameters.

Specimen Number	Sheathing Materials	Number of Sheathing	Sheathing Thickness (mm)	Keel Spacing (mm)
W-400-OS	OSB	1	11	400
W-600-OS	OSB	1	11	600
W-400-OD	OSB	2	11	400
W-600-OD	OSB	2	11	600

**Table 2:** Characteristic values and ductility factor of the specimens.

Specimen Number	Yield Point		Peak Point		Ductility Factor
	*P*_y_ (kN)	Δ_y_ (mm)	*P*_max_ (kN)	Δ_max_ (mm)	*μ*
W-400-OS	32.83	15.07	38.19	16.70	1.11
W-400-OD	65.36	16.92	73.30	20.30	1.20
W-600-OS	34.02	16.40	35.17	18.58	1.13
W-600-OD	61.89	15.72	74.20	18.03	1.15

**Table 3:** The control parameters of the modified exponential “Foschi” skeleton curve.

Control Parameters	*K*_1_ (kN/mm^2^)	*K*_2_ (kN/mm^2^)	*K*_3_ (kN/mm^2^)	*F*_0_ (kN)	*δ_m_* (mm)	*δ_u_* (mm)
selection value	1.869	0.098	−0.125	1.224	6.6	18.6

**Table 4:** Comparison of the results between the test and the fine model analysis.

Specimen	Item	Yield Point		Peak Point		Ductility Factor
		*P*_y_ (kN)	Δ_y_ (mm)	*P*_max_ (kN)	Δ_max_ (mm)	*μ*
W-400-OS	Test	32.83	15.07	38.19	16.70	1.11
	Fine model	29.82	13.19	33.66	17.13	1.29
	Ratio	0.91	0.88	0.88	1.03	1.16
W-400-OD	Test	65.36	16.92	73.30	20.30	1.20
	Fine model	66.30	15.34	75.44	20.02	1.30
	Ratio	1.01	0.91	1.03	0.99	1.08
W-600-OS	Test	34.02	16.40	35.17	18.58	1.13
	Fine model	31.10	14.38	35.56	19.12	1.32
	Ratio	0.91	0.88	1.01	1.03	1.17
W-600-OD	Test	61.89	15.72	74.20	18.03	1.15
	Fine model	61.86	13.92	69.97	18.31	1.29
	Ratio	1.00	0.89	0.94	1.02	1.12

**Table 5:** The comparison between the test results and the simplified model results.

Specimen Number	Item	Yield Point		Peak Point		Ductility Factor
		*P*_y_ (kN)	Δ_y_ (mm)	*P*_max_ (kN)	Δ_max_ (mm)	*μ*
W-400-OS	Test	32.83	15.07	38.19	16.70	1.11
	Simplified model	30.90	12.77	34.63	17.00	1.33
	Ratio	0.94	0.85	0.91	1.02	1.20
W-400-OD	Test	65.36	16.92	73.30	20.30	1.20
	Simplified model	65.71	14.51	73.89	20.01	1.38
	Ratio	1.01	0.86	1.01	0.99	1.15
W-600-OS	Test	34.02	16.40	35.17	18.58	1.13
	Simplified model	32.24	13.83	36.26	19.23	1.37
	Ratio	0.95	0.84	1.03	1.03	1.21
W-600-OD	Test	61.89	15.72	74.20	18.03	1.15
	Simplified model	60.65	13.17	67.98	18.12	1.37
	Ratio	0.98	0.84	0.92	1.00	1.19

**Table 6:** The first three natural vibration periods of the two models.

Model	Mode	Natural Vibration Period (s)	Vibration Mode
Frame without infilled wall	No. 1	1.254	Y
	No. 2	1.139	X
	No. 3	1.086	RotZ
Frame with infilled wall	No. 1	0.867	X
	No. 2	0.541	Y
	No. 3	0.530	RotZ

**Table 7:** Comparison of the lateral displacement of the top floor.

Seismic Wave	Direction	Types	Lateral Displacement (mm)		
			Frame Without Infilled Wall	Frame with Infilled Wall	Reduction Ratio
EL-Centro	X	Frequently	31.57	25.73	18%
	X	Rarely	180.57	139.40	23%
	Y	Frequently	29.83	20.86	30%
	Y	Rarely	170.03	111.80	34%
Taft	X	Frequently	32.91	29.41	11%
	X	Rarely	187.65	133.60	29%
	Y	Frequently	36.49	12.83	65%
	Y	Rarely	208.90	62.05	70%
Chichi-1181	X	Frequently	54.94	35.92	35%
	X	Rarely	247.32	167.90	32%
	Y	Frequently	46.62	13.39	71%
	Y	Rarely	247.57	72.65	71%

**Table 8:** Comparison of the maximal inter-story displacement angle.

Seismic Wave	Direction	Types of Earthquake	Inter-Story Displacement Angle		
			Frame Without Infilled Wall	Frame with Infilled Wall	Reduction Ratio
EL-Centro	X	Frequent	1/381	1/495	23%
	X	Rare	1/66	1/78	15%
	Y	Frequent	1/397	1/613	35%
	Y	Rare	1/70	1/108	35%
Taft	X	Frequent	1/380	1/450	16%
	X	Rare	1/67	1/104	36%
	Y	Frequent	1/381	1/799	52%
	Y	Rare	1/67	1/166	60%
Chichi-1181	X	Frequent	1/251	1/364	31%
	X	Rare	1/58	1/71	18%
	Y	Frequent	1/258	1/781	67%
	Y	Rare	1/53	1/133	60%

**Table 9:** Comparison of the base shear.

Seismic Wave	Direction	Types of Earthquake	Base Shear (KN)		
			Frame Without Infilled Wall	Frame with Infilled Wall	Ratio
EL-Centro	X	Frequent	1339.20	1923.87	+44%
	X	Rare	7622.47	8461.52	+11%
	Y	Frequent	1192.75	2202.11	+85%
	Y	Rare	6759.51	10,026.35	+48%
Taft	X	Frequent	1379.20	1951.27	+41%
	X	Rare	7855.26	7764.40	−1%
	Y	Frequent	1090.74	2393.56	+119%
	Y	Rare	6188.23	9867.01	+59%
Chichi-1181	X	Frequent	2484.19	2318.96	−7%
	X	Rare	9884.89	9354.02	−5%
	Y	Frequent	1615.33	2476.07	+53%
	Y	Rare	8562.47	11,587.39	+35%

## Data Availability

The original contributions presented in this study are included in the article. Further inquiries can be directed to the corresponding author.

## Abbreviations

The following abbreviations are used in this manuscript:

CFS	Cold-formed steel
OSB	Oriented strand board
RCFS	Reinforced cold-formed steel
